# Onset of vitiligo in a psoriasis patient on ixekizumab

**DOI:** 10.1111/dth.15102

**Published:** 2021-09-02

**Authors:** Claudio Marasca, Luigi Fornaro, Fabrizio Martora, Vincenzo Picone, Gabriella Fabbrocini, Matteo Megna

**Affiliations:** ^1^ Section of Dermatology, Department of Clinical Medicine and Surgery University of Naples Federico II Napoli Italy

**Keywords:** anti‐IL‐17, ixekizumab, psoriasis, real life experience, vitiligo

## Abstract

**Introduction:**

Vitiligo is an acquired skin disorder clinically characterized by hypopigmentated macules and patches. Psoriasis is a chronic‐inflammatory‐skin‐condition characterized by erythematous‐plaques covered with scales particularly over the extensor‐surfaces, scalp, and lumbosacral region. Recent major‐researches‐advancements have significantly expanded our understanding of psoriasis‐pathophysiology, resulting in the development of highly effective targeted‐therapies, such as anti TNFα, IL‐12/23‐inhibitors, IL‐17‐inhibitors, or IL‐23‐inhibitors. Particularly, ixekizumab, a humanized‐monoclonal immunoglobulin‐G 4 antibody, specifically binding IL‐17A, demonstrated strong efficacy in threating recalcitrant psoriasis. Nevertheless, paradoxical reactions due to IL‐17 inhibitors have been described.

**Case report:**

Herein, we report the case of a 53‐year‐old Caucasian man who obtained complete skin clearance of psoriasis plaques after 16 weeks of ixekizumab treatment together with the appearance of vitiligo patches localized on the facial area. He had never suffered of vitiligo and his family history excluded vitiligo diagnosis. We also could exclude post inflammatory psoriasis hypopigmentation because of absence of facial involvement at baseline. Our experience suggests that vitiligo might be considered a rare adverse effects of anti‐IL‐17 therapy.

## 1 INTRODUCTION

Vitiligo is an acquired skin disorder clinically characterized by hypopigmentated macules and patches, most frequently localized in periocular area, hands, knees, and genitals caused by the selective autoimmune destruction of melanocytes. The disorder can be psychologically devastating and stigmatizing, especially in dark skinned individuals. It could be associated with other autoimmune disease, such as Hashimoto thyroiditis, diabetes, or other.^1^

Psoriasis is a chronic inflammatory skin condition characterized by erythematous plaques covered with scales particularly over the extensor‐surfaces, scalp and lumbosacral region. Mild clinical manifestations may be controlled with topical agents such as corticosteroids, vitamin D3 analogs, retinoids, calcineurin inhibitors, and keratolytic agents. Systemic treatment are administered for more diffuse forms and include phototherapy (Nb‐UvB), acitretin, methotrexate, or cyclosporine. These treatments may be linked to adverse events or contraindicated in psoriasis patients, which usually reports higher cardio‐metabolic comorbidities respect to general population.^2^

Recent major researches advancements have significantly expanded our understanding of psoriasis pathophysiology, resulting in the development of highly effective targeted therapies, such as anti‐TNFα, IL‐12/23‐inhibitors, IL‐17‐inhibitors, or IL‐23‐inhibitors. Particularly, ixekizumab, a humanized‐monoclonal immunoglobulin‐G 4 antibody, specifically binding IL‐17A, demonstrated strong efficacy in threating recalcitrant psoriasis. Nevertheless, paradoxical reactions due to IL‐17 inhibitors have been described, even for the treatment of hidradenitis suppurativa, but still today, pathophysiology of these events is not so completely understood.^3^, ^4^

Herein, we report the case of a 53‐year‐old Caucasian man who obtained complete skin clearance of psoriasis plaques after 16 weeks of ixekizumab treatment together with the appearance of vitiligo patches localized on the facial area.

## 2 CASE REPORT

A 53‐year‐old Caucasian man was admitted to our outpatient presenting a severe plaque psoriasis, affecting trunk, upper and lower extremities and palms, sparing facial area (PASI 18 BSA 30%). Family history of psoriasis was negative and no comorbidities were recorded. Medical history was unremarkable and the patient did not take any drugs. The patient has been suffering from plaque‐type psoriasis for 20 years, with a chronic remitting course. Clinical lesions were associated with severe discomfort, which impaired daily activities and social relationships. His treatment history has started in 2010 with topical clobetasol propionate 0.05% and calcipotriol with unsatisfactory results. Subsequently, patient was treated with methotrexate that provided adequate efficacy for 52 weeks, when a secondary loss of efficacy was experienced.

**FIGURE 1:**
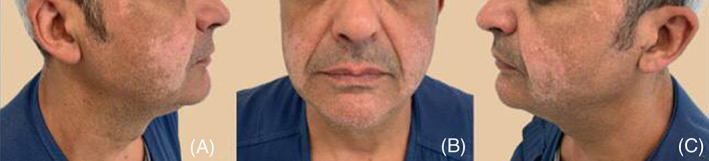
Front side, right side, and left side (A–C)

Therefore, he was switched to adalimumab, leading to an initial optimal response (PASI90). After 7 months clinical improvements were progressively lower with re‐occurrence of clinical lesions. Thereafter, due to the involvement of difficult to treat areas (palms and genital area) and psoriasis worsening (PASI 18 BSA 30%), ixekizumab was started according to the following schedule: 160 mg week 0, 80 mg every 2 weeks for 12 weeks followed by 80 mg every 4 weeks. After only 4 weeks of treatment, we observed a complete skin clearance (PASI 100 response) with a huge improvement in quality of life and skin symptoms. Even if clinical response remained stable over the time, 12 weeks later hypopigmented macules and patches appeared on the facial area, particularly on cheeks and chin (5 cm × 6 cm) saving periocular area. (Figure 1).

Dermatologic examination together with Wood's lamp examination lead to the diagnosis of vitiligo. Blood exams excluded other underlying conditions such as anemia, hyperthyroidism, and diabetes. Treatment with topical calcineurin inhibitors was started. No data regarding follow up are available.

## 3 DISCUSSION

The association between vitiligo and psoriasis has already been reported in literature. Recently, Canu et al. described demographic and clinical characteristics of patients with both psoriasis and vitiligo in a cohort of patients: particularly, of the 436 vitiligo patients included in this study, 74 vitiligo patients (45 women) had a past and/or current personal history of psoriasis. The authors suggested that patients with vitiligo and psoriasis are more prone to a combination of a Th17‐ and Th1‐skewed immune response, referring to the presence in psoriatic skin of memory CD8^+^ T cells directed against the melanocyte‐derived protein ADAMTSL5 that may favor the development of vitiligo in a predisposed patient.^5^

Vitiligo has been reported an idiosyncratic adverse effect of anti‐TNFα agents including adalimumab. TNF‐α is supposed to be as potential cytokine involved in the vitiligo pathogenesis. In fact, anti‐TNF‐α agents have been used in treatment of vitiligo with uncertain results, even if in vivo studies demonstrated that TNF‐α inhibits differentiation of melanocytes from stem cells.^5^ The relationship between psoriasis and melanogenesis, as well as the connection between biologics and vitiligo, has yet to be confirmed. One case of TNFα induced vitiligo, resolved after ixekizumab therapy, has recently been described.^6^

Biologics inhibiting the Th17 pathway have recently been introduced on the market; consequently data about paradoxical adverse effects are not so wide. The most common adverse effects of anti IL17 were nasopharyngitis, upper respiratory tract infections, headache, and arthralgia, which are rarely associated with therapy discontinuation.^7^

Ixekizumab has demonstrated adverse effects comparable with placebo and no severe reactions were described for prolongated treatment (5 years). Adverse effects in course of ixekizumab were similar to other Anti IL‐17 inhibitors.^8^, ^9^

Among others IL 17 inhibitors, Nieto‐Benito et al. reported two cases of secukinumab induced vitiligo. In the first case, the Authors described achromic macules of the dorsum of left foot and of the ventral part of the left arm after 24 months of therapy. In the second case description, the achromic lesions appeared after 12 months of treatment in axillary region without hair involvement.^10^

In addition, it was registered also one case of vitiligo resolution after switching from adalimumab to secukinumab. Vitiligo manifestations were localized on both hands, in the areas where psoriasis patch was originary present.^11^

Moreover, Jerjen reported a case of refractory vitiligo, which was treated with tildrakizumab. After 12 months of therapy, it was observed repigmentation of 90% areas of baseline vitiligo.^12^

In our case, patients developed vitiligo patches on facial area, particularly on cheek and chain. He had never suffered of vitiligo and his family history was also negative for the disease. We also could exclude post inflammatory psoriasis hypopigmentation because of absence of facial involvement at baseline.

While most of the reactions reported have been associated with anti‐TNFα, description of cases related to more recent biologic therapies are increasing worldwide. A multicenter, retrospective study described 18 cases of new‐onset vitiligo under biological treatment for different conditions, including psoriasis. The most involved drugs were: adalimumab followed by ustekinumab (three cases) and secukinumab (one case).^13^

Fortunately, de novo vitiligo in course of biological agents, usually have a better prognosis than conventional or preexisting one and it may be controlled by topical treatment without discontinuation of the drug.

Moreover, PD‐1 inhibitors has also been associated with vitiligo because of their mechanism of action. Vitiligo is a classic side effect of PD‐1 inhibitors, as it has been suggested that melanocytic antigens are released by tumor cells when destroyed by PD‐1 antibodies, which induces an immune response against native melanocytes.

In conclusion, only four cases of de novo vitiligo in course of anti‐IL‐17 monoclonal antibodies have been reported in literature, and ours is the first with ixekizumab. Our experience, although limited to the present case, seems to confirm that vitiligo represents a rare adverse event with using this drug class, which rarely leads to drug interruption. Further studies in a real life setting may support, or not, these data.

## AUTHOR CONTRIBUTION

Claudio Marasca: Have made substantial contributions to conception and design, drafting the manuscript and revising it critically for important intellectual content and given final approval of the version to be published.

Luigi Fornaro: Drafting the article, and revising it critically for important intellectual content and given final approval of the version to be published.

Fabrizio Martora: Drafting the article, bibliography research, acquisition of data, drafting the article.

Vincenzo Picone: Drafting the article, bibliography research, acquisition of data, drafting the article.

Matteo Megna: Have made substantial contributions to conception and design, drafting the article, and revising it critically for important intellectual content and given final approval of the version to be published.

Gabriella Fabbrocini: Have made substantial contributions to conception and design, drafting the manuscript and revising it critically for important intellectual content and given final approval of the version to be published.

## Data Availability

Data sharing not applicable to this article as no datasets were generated or analysed during the current study.
